# Evaluation of T1 relaxation time in prostate cancer and benign prostate tissue using a Modified Look-Locker inversion recovery sequence

**DOI:** 10.1038/s41598-020-59942-z

**Published:** 2020-02-20

**Authors:** Alexander D. J. Baur, Carla M. Hansen, Julian Rogasch, Helena Posch, Sefer Elezkurtaj, Andreas Maxeiner, Katharina Erb-Eigner, Marcus R. Makowski

**Affiliations:** 1 Charité – Universitätsmedizin Berlin, corporate member of Freie Universität Berlin, Humboldt-Universität zu Berlin, and Berlin Institute of Health, Klinik für Radiologie, Augustenburger Platz 1, 13353 Berlin, Germany; 2 Charité – Universitätsmedizin Berlin, corporate member of Freie Universität Berlin, Humboldt-Universität zu Berlin, and Berlin Institute of Health, Klinik für Nuklearmedizin, Augustenburger Platz 1, 13353 Berlin, Germany; 3 Charité – Universitätsmedizin Berlin, corporate member of Freie Universität Berlin, Humboldt-Universität zu Berlin, and Berlin Institute of Health, Institut für Pathologie, Charitéplatz 1, 10117 Berlin, Germany; 4 Charité – Universitätsmedizin Berlin, corporate member of Freie Universität Berlin, Humboldt-Universität zu Berlin, and Berlin Institute of Health, Klinik für Urologie, Charitéplatz 1, 10117 Berlin, Germany; 5 Technische Universität München, Klinikum rechts der Isar, Institut für diagnostische und interventionelle Radiologie, Ismaninger Strasse 22, 81675 Munich, Germany

**Keywords:** Diagnostic markers, Prostate

## Abstract

Purpose of this study was to evaluate the diagnostic performance of T1 relaxation time (T1) for differentiating prostate cancer (PCa) from benign tissue as well as high- from low-grade PCa. Twenty-three patients with suspicion for PCa were included in this prospective study. 3 T MRI including a Modified Look-Locker inversion recovery sequence was acquired. Subsequent targeted and systematic prostate biopsy served as a reference standard. T1 and apparent diffusion coefficient (ADC) value in PCa and reference regions without malignancy as well as high- and low-grade PCa were compared using the Mann-Whitney U test. The performance of T1, ADC value, and a combination of both to differentiate PCa and reference regions was assessed by receiver operating characteristic (ROC) analysis. T1 and ADC value were lower in PCa compared to reference regions in the peripheral and transition zone (p < 0.001). ROC analysis revealed high AUCs for T1 (0.92; 95%-CI, 0.87–0.98) and ADC value (0.97; 95%-CI, 0.94 to 1.0) when differentiating PCa and reference regions. A combination of T1 and ADC value yielded an even higher AUC. The difference was statistically significant comparing it to the AUC for ADC value alone (p = 0.02). No significant differences were found between high- and low-grade PCa for T1 (p = 0.31) and ADC value (p = 0.8). T1 relaxation time differs significantly between PCa and benign prostate tissue with lower T1 in PCa. It could represent an imaging biomarker for PCa.

## Introduction

Magnetic resonance imaging (MRI) has proven its potential to detect clinically significant prostate cancer (PCa) with high accuracy and provide information regarding local tumor staging^1–4^. According to current guidelines, multiparametric MRI (mpMRI) combining high-resolution T2-weighted imaging (T2WI), diffusion-weighted imaging (DWI), and dynamic contrast-enhanced (DCE) MRI should be evaluated semi-quantitatively using the prostate imaging - reporting and data system (PI-RADS)^5^. In addition, DWI and DCE can be evaluated quantitatively, and selected derived parameters have proven promising imaging biomarkers for the characterization of cancerous tissues within the prostate^6–9^.

Non-contrast-enhanced T1-weighted imaging (T1WI) currently only is used for detection of hemorrhage and no information regarding tumor characterization is derived from this sequence type^5,10^. T1 mapping enables the reliable evaluation of the spin-lattice relaxation time (T1) and thus provides reproducible data on intrinsic tissue values^11^. It can be used as a fast, quantitative and noninvasive technique to determine biological tissue properties^12^. In prostate MRI, non-contrast-enhanced T1 mapping is used for computation of parametric pharmacokinetic maps from signal-intensity curves acquired during DCE. In the context of cardiovascular imaging, T1 mapping has been established as an essential tool for characterization of myocardial tissue including the quantification of amyloidosis and detection of inflammatory myopathy^13,14^. Few studies have evaluated its value for prostate imaging with mixed results using different technical approaches^15–17^.

The purpose of this explorative study was to evaluate if non-contrast-enhanced T1 relaxation time (T1) measured using a Modified Look-Locker inversion recovery sequence (MOLLI) differs between malignant prostate lesions and tissue not harboring malignancy as well as high- and low-grade PCa and thus could be used as an imaging biomarker for the detection of PCa and the prediction of tumor grading.

## Material and Methods

### Study population

For this prospective study 90 consecutive patients with clinical suspicion for PCa were screened between July 2016 and December 2018. Patients with general contraindications for MRI, for prostate biopsy, and patients with previously biopsy-proven PCa were excluded. Only patients who subsequently underwent prostate biopsy at our institution were included in the final evaluation. Our institutional ethics review board (Ethikkommission der Charité – Universitätsmedizin Berlin) approved this study (application number EA1/076/16). Written informed consent was obtained from all participants. All research was performed in accordance with relevant guidelines/regulations.

### Acquisition of MRI and T1 mapping

Before undergoing biopsy, all patients were examined with a single 3 T MRI scanner (Magnetom Skyra, Siemens, Erlangen, Germany). Biparametric MRI including T2WI and DWI was performed using an imaging protocol in accordance with recommendations from current guidelines^5^.

Sequence parameters of axial echo-planar imaging DWI were as follows: field-of-view 220*220 mm, in-plane resolution 1.4*1.4 mm, slice thickness 3 mm, no gap, 23 slices, repetition time (TR) 4,400 ms, echo time (TE) 61 ms, measured b-values of 50, 100, 500, and 1,000 s/mm^2^, total acquisition time 5 min 5 s. ADC maps were calculated based on b-values of 50 and 1,000 s/mm^2^ and images with a b-value of 1,400 s/mm^2^ were also calculated by the software preinstalled on the MRI scanner (software version VE11A). For T1 mapping MOLLI was used^11^. In this sequence, radio frequency pulses are applied to cause an inversion of the protons’ spins. Within defined time intervals (inversion time), serial T1WI is acquired. Images were acquired sequentially for each slice and sequence parameters were as follows: field-of-view 281*281 mm, in-plane resolution 1.3*1.3 mm, slice thickness 3 mm, no gap, 20 slices, TR 808.6 ms, TE 2.45 ms, flip-angle 35°, total acquisition time 4 min 28 s. T1 maps were calculated by the software preinstalled on the MRI scanner (see above).

All patients were asked to empty their rectum before the examination. However, no mechanical evacuation of air from the rectum was performed.

### Evaluation of MRI

Biparametric MRI was evaluated by an uroradiologist with more than 5 years of experience in reporting of mpMRI of the prostate and classified according to PI-RADS version 2^5^. Suspicious or equivocal lesions (PI-RADS ≥3) were identified and marked on a standardized sector map.

### Prostate biopsy

Patients included in the final evaluation underwent prostate biopsy at our institution after MRI in order to assure that all biopsies were performed using the same technical approach and according to the same standard. All but one patient underwent systematic transrectal ultrasound (TRUS)-guided biopsy. In patients with suspicious or equivocal lesions (PI-RADS ≥3) additional targeted biopsies of these lesions were acquired using an MRI/TRUS fusion technique already described elsewhere^18^. One patient with a suspicious lesion refused to undergo systematic transrectal biopsy and only underwent targeted direct MRI-guided in-bore biopsy of the respective lesion.

### Histopathological evaluation

All biopsy cores from systematic and targeted biopsies were evaluated. Gleason patterns and grade groups were defined according to the recommendations of the 2014 International Society of Urological Pathology (ISUP) consensus conference on Gleason grading of prostatic carcinoma^19^. The cores with the highest ISUP grade group from targeted biopsies or systematic biopsies taken from the area of a suspicious or equivocal lesion identified on MRI were used as a reference standard for each respective lesion.

### Evaluation of T1

T1 maps were evaluated by a single uroradiologist with more than 5 years of experience in reporting of mpMRI of the prostate blinded to the results of biopsy using a dedicated software (Visage 7, Version 7.1.11, Visage Imaging GmbH, Berlin, Germany). On axial imaging a circular region-of-interest (ROI) was placed within each suspicious or equivocal lesion previously identified on T2WI and DWI and subsequently targeted by biopsy. The size of each ROI was adapted to the size of the respective lesion. ROIs were placed within each lesion on the sequence best depicting the lesion (T2WI or DWI) and then copied to the same location on the other sequences including T1 maps. Manual corrections were made, if there was an obvious misalignment between the sequences due to patient or bowel movement or image distortion occurring on DWI. Within these ROIs mean T1 (ms) and apparent diffusion coefficient (ADC) value (mm^2^/s) were measured.

**Figure 1:**
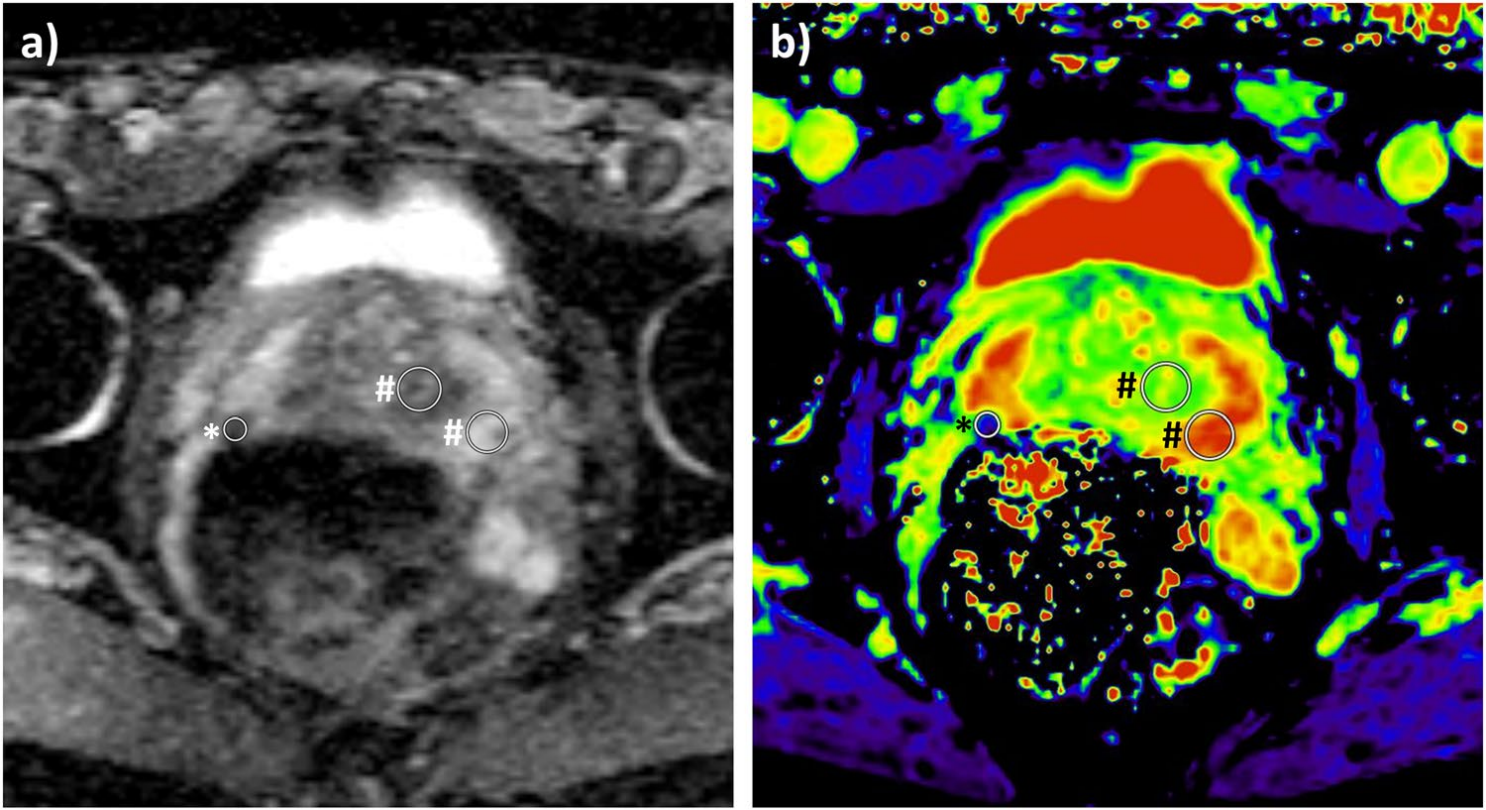
Exemplary images depicting placement of ROIs in a PI-RADS 4 lesion in the PZ (marked by asterisk) as well as contralateral reference regions in the PZ and TZ at the level of the base of the prostate (marked by octothorpes) on (**a**) the ADC map and (**b**) the color-coded T1 map with red representing a high T1 and blue representing a low T1 in a 64-year-old patient. Targeted biopsy of the PI-RADS 4 lesion yielded ISUP grade group 2 PCa whilst systematic biopsy yielded no PCa in the contralateral reference regions.

In addition, mean T1 and ADC value were measured within circular ROIs with a minimum size of 50 mm^2^ in the peripheral zone (PZ) and transition zone (TZ) in areas not suspicious on biparametric MRI (PI-RADS score <3) at the level of the apex, the midgland, and the base on both sides of each patient’s prostate who had undergone systematic biopsy. Measurements within these ROIs served as a reference for non-cancerous tissue. Measurements in areas surrounding suspicious or equivocal lesions identified on MRI or in areas with biopsy-proven PCa on systematic biopsy without a corresponding lesion on MRI were omitted. For exemplary placement of ROIs see Fig. 1.

### Statistical evaluation

T1 and ADC value of suspicious or equivocal lesions identified on biparametric MRI (PI-RADS score ≥3; lesions) were compared to T1 and ADC value of regions not suspicious on biparametric MRI (PI-RADS score <3) without corresponding biopsy-proven PCa on systematic biopsy (reference regions). Regions that yielded PCa on systematic biopsy whilst not being suspicious on biparametric MRI (PI-RADS score <3) were excluded from further evaluation. This was done as due to the lack of a circumscribed lesion on imaging and the impossibility to exactly define the location from which the respective biopsy core was taken during systematic biopsy, it would not have been possible to ensure that T1 and ADC value measured within a ROI would have been representing the biopsy-proven PCa or surrounding benign tissue. In the one patient who only underwent targeted direct MRI-guided in-bore biopsy only measurements within the target lesion were performed.

In addition, T1 and ADC value of suspicious or equivocal lesions identified on biparametric MRI (PI-RADS score ≥3) with biopsy-proven PCa (PCa lesions) were compared to T1 and ADC value of regions not suspicious on biparametric MRI (PI-RADS score <3) without corresponding biopsy-proven PCa on systematic biopsy (reference regions). As a result, suspicious or equivocal lesions on biparametric MRI (PI-RADS score ≥3) without corresponding biopsy-proven PCa were excluded from any further evaluation to exclude PCa potentially missed by targeted biopsy.

In order to evaluate differences in T1 and ADC value between clinically significant and not clinically significant PCa, PCa lesions were grouped into lesions with an ISUP grade group ≥3 (considered clinically significant) as well as lesions with an ISUP grade group <3 (considered not clinically significant). Based on the Shapiro-Wilk test non-normal distribution of the data was assumed. Therefore, statistical analysis was performed with the package ‘*clusrank*’ for R 3.5.1 (Foundation for Statistical Computing, Vienna, Austria, 2018, http://www.R-project.org) using the Mann-Whitney U test with correction for clustered data (patients) according to Rosner *et al*.^20^. A two-sided p-value ≤ 0.05 was considered statistically significant.

Receiver operating characteristic (ROC) analysis to assess the performance of T1 and ADC value to differentiate between PCa lesions and reference regions was performed using the nonparametric (empirical) approach for clustered data by Obuchowski *et al*.^21^. Calculations were performed using the script ‘funcs_clusteredROC.R’ for R developed by the Lerner Research Institute (Cleveland Clinic, Cleveland, OH, USA) and made publicly available by the developers (https://www.lerner.ccf.org/qhs/software/lib/funcs_clusteredROC.R). The resulting areas under the curve (AUC) were compared using the method by Obuchowski *et al*. Cut-off analysis was done using Youden’s Index^22^. In addition, ROC analysis was performed using binarized data for T1 and ADC value (low versus high T1 and ADC value based on the optimal cut-offs) as well as a combination of T1 and ADC value (T1 and ADC value both low, T1 or ADC value low, T1 and ADC value high).

ROC curves were generated using SPSS Version 22 (IBM, Chicago, IL, USA).

## Results

Out of 90 patients screened for this study 23 patients subsequently underwent biopsy of the prostate at our institution and were included in the final evaluation. Median age was 71 years (range, 55 to 79 years), median prostate-specific antigen (PSA) was 6.98 ng/ml (range, 3.54 to 18.6 ng/ml). On biparametric MRI the most suspicious lesion was a PI-RADS 5 lesion in 8 patients, a PI-RADS 4 lesion in 9 patients and a PI-RADS 3 lesion in 1 patient. PI-RADS 2 lesions were seen in 5 patients. In 3 of the patients with a suspicious or equivocal lesion an additional suspicious or equivocal lesion was identified on biparametric MRI (a PI-RADS 5 lesion in 2 patients and a PI-RADS 4 lesion in 1 patient).

Of the 21 suspicious or equivocal lesions (PI-RADS ≥3) 15 were located in the PZ and 6 were located in the TZ. Median time between MRI and biopsy was 43 days (range, 2 to 188 days). In patients with suspicious or equivocal lesions (PI-RADS ≥3) who underwent systematic TRUS biopsy plus additional targeted biopsies of these lesions median time between MRI and biopsy was 44 days (range, 2 to 79 days). A median number of 2 targeted biopsy cores were taken from each suspicious or equivocal lesion (range, 2 to 5). In one patient with a PI-RADS 4 lesion targeted MRI/TRUS fusion biopsy did not yield PCa. Due to the high suspicion for PCa on biparametric MRI an additional direct MRI-guided in-bore biopsy was performed and yielded PCa previously missed by MRI/TRUS fusion biopsy.

In total, in 17 out of 21 suspicious or equivocal lesions targeted biopsy yielded PCa (PCa lesions). Median lesion diameter of PCa lesions was 16 mm (range, 6 to 25 mm) and median lesion diameter of lesions that did not yield PCa was 9 mm (range, 7 to 24 mm). Nine lesions were ISUP grade group 1 PCa, 5 lesions were ISUP grade group 2 PCa, and 3 lesions were ISUP grade group 4 PCa. 4 out of 21 suspicious or equivocal lesions did not yield PCa in biopsy.

In 5 patients systematic biopsy yielded ISUP grade group 1 PCa in areas not suspicious on biparametric MRI. These areas were excluded from further evaluation and not used as reference regions. A total of 212 reference regions were included of which 106 were located in the PZ and 106 were located in the TZ.

For median T1 and ADC value in lesions, PCa lesions, and reference regions please see Tables 1 and 2. T1 and ADC value were significantly lower in reference regions in the TZ compared to the PZ (p < 0.001). T1 and ADC value of lesions and PCa lesions were significantly lower than T1 and ADC value of reference regions in the whole prostate (PZ and TZ combined, p < 0.001) and T1 and ADC value of lesions and PCa lesions subdivided by PZ and TZ were significantly lower than T1 and ADC value of the corresponding reference regions (PZ or TZ, each p < 0.001). When comparing T1 and ADC value of PCa lesions with an ISUP grade group ≥3 (n = 3) and PCa lesions with an ISUP grade group <3 (n = 14), no statistically significant differences were found (T1, p = 0.31; ADC, p = 0.8). For T1 and ADC values of PCa lesions within different ISUP grade groups please see Table 3.

**Table 1:** T1 and ADC value in lesions and PCa lesions.

Lesions						PCa lesions					
All (n = 21)		PZ (n = 15)		TZ (n = 6)		All (n = 17)		PZ (n = 12)		TZ (n = 5)	
T1 in ms; median (range)	ADC values in mm^2^/s; median (range)	T1 in ms; median (range)	ADC values in mm^2^/s; median (range)	T1 in ms; median (range)	ADC values in mm^2^/s; median (range)	T1 in ms; median (range)	ADC values in mm^2^/s; median (range)	T1 in ms; median (range)	ADC values in mm^2^/s; median (range)	T1 in ms; median (range)	ADC values in mm^2^/s; median (range)
1,328 (1,037–1,532)	796 (580–1,195)	1,342 (1,037–1,532)	930 (679–1,195)	1,243 (1,192–1,341)	701 (580–789)	1,301 (1,037–1,532)	789 (580–1,195)	1,332 (1,037–1,532)	943 (679–1,195)	1,247 (1,192–1,341)	689 (580–789)

T1 and ADC value of lesions (lesions with PI-RADS ≥3) and PCa lesions (lesions with PI-RADS ≥3 and biopsy-proven PCa) in the whole prostate, peripheral zone, and transition zone. ADC, apparent diffusion coefficient; PCa, prostate cancer; PZ, peripheral zone; T1, T1 relaxation time; TZ, transition zone.

**Table 2:** T1 and ADC value in reference regions.

	PZ (reference regions; n = 106)		TZ (reference regions; n = 106))	
	T1 in ms; median (range)	ADC values in mm^2^/s; median (range)	T1 in ms; median (range)	ADC values in mm^2^/s; median (range)
Base	1,666 (1,222–2,343)	1,502 (1,084–1,979)	1,486 (1,301–1,757)	1,190 (851–1,560)
Midgland	1,756 (1,488–2,412)	1,586 (1,192–1,957)	1,503 (1,343–1,799)	1,161 (919–1,451)
Apex	1,759 (1,083–2,351)	1,569 (1,076–1,976)	1,508 (1,037–1,756)	1,261 (914–1,811)

T1 and ADC value of reference regions (PI-RADS <3 and negative histopathology) in the peripheral zone and transition zone. ADC, apparent diffusion coefficient; PZ, peripheral zone; T1, T1 relaxation time; TZ, transition zone.

**Table 3:** T1 and ADC value in PCa lesions separated by ISUP grade groups.

ISUP grade group 1 (n = 9)		ISUP grade group 2 (n = 5)		ISUP grade group 4 (n = 3)	
T1 in ms; median (range)	ADC value in mm^2^/s; median (range)	T1 in ms; median (range)	ADC value in mm^2^/s; median (range)	T1 in ms; median (range)	ADC value in mm^2^/s; median (range)
1,336 (1,238–1,532)	955 (580–1,195)	1,199 (1,037–1,341)	689 (587–930)	1,247 (1,223–1,344)	789 (715–1,056)

T1 and ADC value of PCa lesions (lesions with PI-RADS ≥3 and biopsy-proven PCa) separated by ISUP grade groups. ADC, apparent diffusion coefficient; ISUP, International Society of Urological Pathology; PCa, prostate cancer; T1, T1 relaxation time.

**Figure 2:**
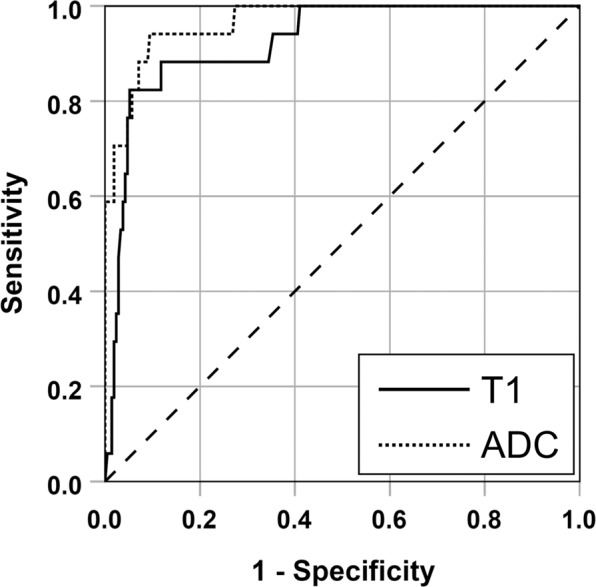
ROC curves for T1 and ADC value when differentiating PCa lesions and reference regions in the whole prostate (PZ and TZ combined).

ROC analysis showed high AUCs for T1 (0.92; 95%-confidence interval [95%-CI], 0.87–0.98) and ADC value (0.97; 95%-CI, 0.94 to 1.0) when differentiating PCa lesions and reference regions in the whole prostate (PZ and TZ; see Fig. 2), and this AUC was significantly higher for ADC value than for T1 (AUC, 0.97 versus 0.92; 95%-CI of the AUC difference, 0.005 to 0.09; p = 0.03). AUCs for T1 and ADC value were even higher when differentiating PCa lesions in PZ and TZ from reference regions in the PZ and TZ separately and were significantly different in the PZ (PZ: T1: 0.95; 95%-CI, 0.91–0.99; ADC value: 0.99; 95%-CI, 0.98–1.0; p = 0.03; TZ: T1: 0.97; 95%-CI, 0.93–1.0; ADC value: 1.0; 95%-CI, 1.0–1.0; p = 0.25). For optimal cut-off values for T1 and ADC value and resulting sensitivities and specificities for the whole prostate please see Table 4.

**Table 4:** Optimal cut-offs for T1 and ADC values.

	Optimal cut-off	TP	FN	TN	FP	Sensitivity (95%-CI)	Specificity (95%-CI)
T1	≤1,345 ms	14	3	201	11	82% (57–96%)	95% (91–97%)
ADC value	≤1,078 mm^2^/s	16	1	192	20	94% (71–100%)	90% (85–94%)
T1 or ADC value	*see above*	16	1	185	27	94% (71–100%)	87% (82–91%)
T1 and ADC value	*see above*	14	3	208	4	82% (57–96%)	98% (95–100%)

Optimal cut-offs as well as resulting sensitivities and specificities for T1 and ADC values to differentiate 17 PCa lesions and 212 reference regions (4 lesions with discordant findings on MRI and biopsy were excluded). Sensitivity and specificity of a combination of binarized T1 and ADC value are also given – rating either a low T1 or ADC value or only the combination of both low values for both parameters as positive. ADC, apparent diffusion coefficient; CI, confidence interval; T1, T1 relaxation time; TP, true positives; FN, false negatives; TN, true negatives; FP, false positives.

Using binarized data for T1 and ADC value when differentiating PCa lesions and reference regions in the whole prostate ROC analysis also revealed high AUCs for T1 (0.89; 95%-CI, 0.79–0.98) and ADC value (0.92; 95%-CI, 0.85–0.99) with a combination of T1 and ADC value showing an even higher AUC of 0.95 (95%-CI, 0.88–1.0). Comparing AUCs for ADC value and a combination of T1 and ADC value statistically significant differences were found (0.92 versus 0.95, p = 0.02) whilst comparing T1 and a combination of T1 and ADC value no statistically significant differences were found (0.89 versus 0.95, p = 0.07).

## Discussion

T1 was significantly lower in lesions and PCa lesions compared to reference regions in the PZ and TZ of the prostate (p < 0.001, respectively). However, no statistically significant differences for T1 and ADC value could be shown between high- and low-grade PCa.

PI-RADS is a structured reporting system for prostate mpMRI based on the semi-quantitative evaluation of T2WI, DWI, and DCE MRI^5^. One of the major limitations of PI-RADS is its limited interreader agreement, even between experienced readers^23^. The quantitative evaluation of MRI sequences as imaging biomarkers as well as the use of predefined cut-off values could help to overcome the limitation of a limited interreader agreement and also help to predict tumor grading. The value of the ADC value^24–27^ as well as of several parameters derived from DCE MRI^8,9,28^ has been demonstrated in several studies. However, a quantitative evaluation of these MRI sequences is currently not part of PI-RADS^5^.

The evaluation of prostate tissue on T2WI is an integral part of PI-RADS^5^. Applying the technical specifications for sequence acquisition of PI-RADS T2WI is acquired in a way that it cannot be evaluated quantitatively. However, in selected studies T2 relaxation time has been shown to differ significantly between malignant and benign prostate lesions as well as high- and low-grade PCa and therefore represents an imaging biomarker that is able to detect PCa and help predict its grading^29–31^. T1WI currently only plays a minor role in the interpretation of prostate mpMRI. It is used primarily to detect hemorrhage within the prostate and seminal vesicles. No relevant information regarding tumor characterization is derived from this sequence type. T1 mapping is an emerging quantitative diagnostic tool in the field of MRI that enables the direct visualization and quantification of the T1 of tissues. To generate a T1 map, proton spin-lattice relaxation times are calculated for each voxel using multiple raw images with different degrees of recovery of longitudinal or spin-lattice magnetization.

Currently, in prostate MRI non-contrast-enhanced T1 mapping is most frequently used for computation of parametric pharmacokinetic maps from signal-intensity curves acquired during DCE MRI. Techniques using at least two acquisitions with different flip angles are commonly used for this purpose^32^ but other techniques have also been evaluated in order to further optimize the accuracy of T1 mapping^33^. Furthermore, pathological tissue alterations as a result of disease processes such as extracellular matrix expression and tissue fibrosis can be noninvasively visualized and quantified based on changes in T1 relaxation properties^13,14,34,35^. Few studies have already evaluated non-contrast-enhanced T1 mapping for the diagnosis of PCa using different technical approaches. In 1987, Kjaer *et al*. were not able to detect significant differences in T1 between PCa and benign prostate hyperplasia in 18 patients using six inversion recovery sequences with a fixed TR and different inversion times (TI) and a 1.5 T magnet^15^. Foltz *et al*. found significantly shorter T1 in PCa lesions compared to normal appearing prostate tissue in 13 patients using a magnetization-prepared spiral technique and a 1.5 T magnet^16^. More recently, Ma *et al*. used MR fingerprinting^36^ and a 3 T magnet to quantitatively measure T1 on non-contrast-enhanced MRI in 109 PCa lesions and normal appearing prostate tissue in the PZ^17^. They found T1 to differ significantly between PCa and normal appearing prostate tissue but failed to show significant differences between high- and low-grade PCa. However, they only evaluated lesions and reference regions in the PZ. Their results for the PZ are in line with our results, with overall shorter T1 in our study in PCa lesions (1,301 ms compared to 1,628 ms) as well as in reference regions (1,756 ms at the level of the midgland compared to 2,247 ms)^17^.

The inversion recovery based modified Look-Locker imaging sequence (MOLLI) is currently the most widely used and among the best validated sequences for T1 mapping^11^. It has been used in DCE MRI as a T1 mapping technique for computation of parametric pharmacokinetic maps and has proven an accuracy and repeatability rated clinically acceptable in phantom studies^37^. To our best knowledge, any further use of MOLLI for prostate imaging has not been reported yet. T1 measured with MOLLI has proven to be a robust imaging biomarker in cardiac MRI^13,14^ as well as selected malignancies such as renal cell cancer with an excellent interreader agreement^35^.

In this explorative study we evaluated T1 measured on non-contrast-enhanced MRI using MOLLI as an imaging biomarker for PCa. We were able to show that T1 and ADC value of the PZ and TZ differ significantly from each other and that they differ from T1 and ADC value of PCa in either zone. This allowed to distinguish PCa lesions from reference regions in the prostate with high AUCs, sensitivity, and specificity. A combination of T1 and ADC value yielded an even higher AUC compared to the AUCs of T1 and ADC value alone. This difference was found to be statistically significant when comparing a combination of T1 and ADC value with ADC value (p = 0.02). However, due to the explorative character of the current analysis, sensitivity and specificity should be regarded with caution as the underlying cut-off values are optimized for the present patient sample. Therefore, cut-off values are potentially biased, and sensitivity and specificity may be overestimated. We were not able to demonstrate any statistically significant differences between T1 and ADC value in high- and low-grade PCa. Other studies have shown that ADC values do differ statistically significantly between high- and low-grade PCa^24–27^. Such differences might have simply been underestimated in our study due to the small number of included PCa lesions as well as the small number of high-grade PCa lesions (n = 3). However, in line with our results Yu *et al*. were also not able to demonstrate differences in T1 between high- and low-grade PCa using a different technique for mapping^17^.

The small number of patients and PCa lesions included is a major limitation of this explorative study. A further limitation is that target lesions for biopsy and evaluation of T1 were previously identified based on T2WI and DWI using PI-RADS version 2. Therefore, no statement can be made about the performance of T1 in PCa occult or presenting with only minimal signal changes on T2WI or DWI as well as the dependence or independence of T1 from signal alterations on T2WI and DWI. Furthermore, not all biopsies of equivocal or suspicious lesions on biparametric MRI yielded PCa and this could have been due to the absence of PCa. On the other hand, PCa might have been missed by targeted biopsy. Therefore, further evaluations focused on PCa lesions. This has led to a selection bias potentially overestimating the value of T1. However, inclusion of the abovementioned lesions as benign tissues could also have led to a bias due to PCa lesions missed by targeted biopsy, as illustrated by one of the patients included in this study where initial targeted MRI/TRUS fusion biopsy missed the correct diagnosis and only subsequent targeted direct MRI-guided in-bore biopsy yielded PCa. In order to evaluate the value of T1 independently from other MRI sequences ideally whole-mount prostatectomy specimens should be used as a reference standard. Finally, the histopathological changes that result in the observed differences in T1 in PCa lesions and reference regions currently remain unclear.

In conclusion, in this explorative study T1 mapping using MOLLI was able to differentiate PCa lesions from prostatic reference regions not harboring malignancy with lower T1 in PCa lesions. Further evaluation in larger patient cohorts is necessary in order to confirm our results, evaluate if T1 determined by MOLLI allows to differentiate between malignant and benign lesions as well as high- and low-grade PCa, and provides added value to the sequences routinely used for prostate MRI.

## Data Availability

The datasets generated and analyzed during the current study are available from the corresponding author on reasonable request.
