# Author Correction: Evaluation of T1 relaxation time in prostate cancer and benign prostate tissue using a Modified Look-Locker inversion recovery sequence

**DOI:** 10.1038/s41598-020-61549-3

**Published:** 2020-03-18

**Authors:** Alexander D. J. Baur, Carla M. Hansen, Julian Rogasch, Helena Posch, Sefer Elezkurtaj, Andreas Maxeiner, Katharina Erb-Eigner, Marcus R. Makowski

**Affiliations:** 1 Charité – Universitätsmedizin Berlin, corporate member of Freie Universität Berlin, Humboldt-Universität zu Berlin, and Berlin Institute of Health, Klinik für Radiologie, Augustenburger Platz 1, 13353 Berlin, Germany; 2 Charité – Universitätsmedizin Berlin, corporate member of Freie Universität Berlin, Humboldt-Universität zu Berlin, and Berlin Institute of Health, Klinik für Nuklearmedizin, Augustenburger Platz 1, 13353 Berlin, Germany; 3 Charité – Universitätsmedizin Berlin, corporate member of Freie Universität Berlin, Humboldt-Universität zu Berlin, and Berlin Institute of Health, Institut für Pathologie, Charitéplatz 1, 10117 Berlin, Germany; 4 Charité – Universitätsmedizin Berlin, corporate member of Freie Universität Berlin, Humboldt-Universität zu Berlin, and Berlin Institute of Health, Klinik für Urologie, Charitéplatz 1, 10117 Berlin, Germany; 5 Technische Universität München, Klinikum rechts der Isar, Institut für diagnostische und interventionelle Radiologie, Ismaninger Strasse 22, 81675 Munich, Germany

Correction to: *Scientific Reports* 10.1038/s41598-020-59942-z, published online 20 February 2020

The Acknowledgements section in this Article was omitted. The Acknowledgements section should read:

“We acknowledge support from the German Research Foundation (DFG) and the Open Access Publication Funds of Charité – Universitätsmedizin Berlin.”

